# Development of a dry powder formulation for pulmonary delivery of azithromycin-loaded nanoparticles

**DOI:** 10.3389/jpps.2024.13635

**Published:** 2024-10-14

**Authors:** Alison Tatiana Madrid Sani, Khellida Loiane V. Ramos-Rocha, Michelle Alvares Sarcinelli, Marcelo Henrique da Cunha Chaves, Helvécio Vinícius Antunes Rocha, Patrícia Léo, Natália Neto Pereira Cerize, Maria Helena Ambrosio Zanin, Valker Araujo Feitosa, Carlota de Oliveira Rangel-Yagui

**Affiliations:** ^1^ Bionanomanufacturing Center, Technological Research Institute, São Paulo, Brazil; ^2^ Department of Biochemical and Pharmaceutical Technology, University of São Paulo, São Paulo, Brazil; ^3^ Laboratory of Micro and Nanotechnology, Fundação Oswaldo Cruz, Rio de Janeiro, Brazil

**Keywords:** spray-drying, polycaprolactone, azithromycin, dry powder inhaler, pulmonary diseases

## Abstract

The COVID-19 pandemic has raised concern regarding respiratory system diseases and oral inhalation stands out as an attractive non-invasive route of administration for pulmonary diseases such as chronic bronchitis, cystic fibrosis, COVID-19 and community-acquired pneumonia. In this context, we encapsulated azithromycin in polycaprolactone nanoparticles functionalized with phospholipids rich in dipalmitoylphosphatidylcholine and further produced a fine powder formulation by spray drying with monohydrated lactose. Nanoparticles obtained by the emulsion/solvent diffusion-evaporation technique exhibited a mean hydrodynamic diameter around 195–228 nm with a narrow monomodal size distribution (PdI < 0.2). Nanoparticle dispersions were spray-dried at different inlet temperatures, atomizing air-flow, aspirator air flow, and feed rate, using lactose as a drying aid, resulting in a maximal process yield of 63% and an encapsulation efficiency of 83%. Excipients and the dry powder formulations were characterized in terms of morphology, chemical structure, thermal analyses and particle size by SEM, FTIR, DSC/TGA and laser light diffraction. The results indicated spherical particles with 90% at 4.06 µm or below, an adequate size for pulmonary delivery. Aerosolization performance in a NGI confirmed good aerodynamic properties. Microbiological assays showed that the formulation preserves AZM antimicrobial effect against *Staphylococcus aureus* and *Streptococcus* pneumoniae strains, with halos above 18 mm. In addition, no formulation-related cytotoxicity was observed against the human cell lines BEAS-2B (lung epithelial), HUVEC (endothelial) and HFF1 (fibroblasts). Overall, the approach described here allows the production of AZM-PCL nanoparticles incorporated into inhalable microparticles, enabling more efficient pulmonary therapy of lung infections.

## Introduction

Respiratory diseases, such as chronic bronchitis, tuberculosis, cystic fibrosis, obstructive pulmonary disease (COPD), COVID-19 and community-acquired pneumonia (CAP) mainly involve the lungs and represent the second cause of death in the world [1]. In particular, CAP refers to pneumonia occurring outside of a hospital and may result from serious respiratory tract infections caused by bacteria, fungus or viruses in children and older adult [2, 3]. The most common treatment for bacterial respiratory infections is the oral administration of high doses of antibiotics, in particular azithromycin (AZM) [4], a macrolide with a broad antibacterial spectrum and high activity against gram-negative and gram-positive organisms [5]. It also presents anti-inflammatory and immunomodulatory properties. Nonetheless, oral administration of AZM can cause nausea, vomiting, diarrhea, and abdominal pain, especially in children [6]. The most common adverse effects associated with AZM involve the gastrointestinal tract and the central and peripheral nervous system [7]. In addition, we can mention severe dizziness, headache, fatigue, and cutaneous manifestations. Less common but serious side effects such as liver damage occur because AZM has become commonly used [8]. Abnormal heart rhythm changes including ventricular arrhythmias may also occur owing to the potential to prolong the QT interval [9]. In some rare cases AZM is associated with an increased risk of cardiovascular death [10]. Therefore, alternative routes of administration such as the pulmonary could benefit patients treated with AZM.

The inhalation route has several advantages when compared to the oral and parenteral routes such as avoiding first-pass hepatic metabolism, rapid diffusion in the lower airways, faster local therapeutic action and higher efficacy, lower doses, and therefore, less direct exposure of other organs to the drug and reduction of side effects [11]. Dry powder inhalers (DPIs) represent the most promising devices for pulmonary administration of drugs. They are safer to the environment since propellant gases are not used and are relatively easy to use by the population [12]. The success of this type of formulation, i.e., maximal deposition in the lower airways, depends on the dry powder properties such as composition, size (below 5 µm), surface morphology and aggregation [13]. A rapid, economic and scalable technique to produce adequate dry powders refers to spray drying, a process in which a dispersion is converted into small droplets to be dried in a hot air chamber [14, 15]. With the proper selection of process conditions, a homogenous powder with optimal particle characteristics can be obtained.

In this work, AZM-loaded nanoparticles of polycaprolactone (AZM-PCL-NPs) functionalized with phospholipids rich in dipalmitoylphosphatidylcholine were prepared by emulsion/solvent diffusion-evaporation technique and the colloidal dispersion was further used to produce AZM-loaded microparticles of polycaprolactone (AZM-PCL-MPs), with lactose as a drying aid, using spray-drying process, resulting in a final dry powder formulation of nano-in-microparticles with adequate size for pulmonary delivery.

## Materials and methods

### Preparation of AZM-PCL nanoparticles (NPs)

AZM-PCL-NP were produced by emulsion/solvent diffusion-evaporation technique. This method involves three main steps, emulsification 1): both the drug and the encapsulating polymer are dissolved in an organic solvent followed by homogenization in an aqueous solution with a dispersing agent forming nanometric droplets. The next step involves diffusion 2): solvent diffuses from the organic phase of the emulsion by the addition of water in excessive quantities resulting in the precipitation of the polymer in the droplets thus producing a dispersion of NPs. Further, the dispersion is subjected to evaporation 3): the solvent is removed under reduced pressure resulting in an aqueous dispersion of NPs.

In detail, the organic phase containing AZM dihydrate (Pliva, Croatia), PCL (10 kDa, Sigma Aldrich, United States) and soybean lecithin S75^®^ (phospholipids rich in dipalmitoylphosphatidylcholine-DPPC, Lipoid, Germany), from now referred as DPPC, were dissolved in 25 mL of ethyl acetate in an ultrasound bath (P30 kHz, Elma, Germany) at 50°C during 25 min in the molar ratio of AZM:PCL:DPPC (0.06, 0.19, 0.75). This phase was added slowly to 100 mL aqueous solution of Pluronic^®^ F127 (10 mg/mL) (EO/PO/EO triblock copolymer, 80% EO, 8400 MW, Sigma Aldrich, United States) and emulsified by 5 min at 14,000 rpm in an Ultra-turrax^®^ (IKA, Germany). Then, 150 mL deionized water was added as a dilution phase, allowing the diffusion of ethyl acetate from the organic phase to the aqueous phase. The resulting dispersion was evaporated at 50°C, 100 rpm, under reduced pressure (45 mbar) in a rotary evaporator R-215 (Büchi, Switzerland) to remove the organic solvent, resulting in AZM-PCL-NP formulations. Furthermore, placebo nanoparticles (PCL-NP) were prepared for comparison purposes.

### Characterization and stability of the nanoparticles

The PCL-NP and AZM-PCL-NP dispersions were stored in a refrigerator (4°C) and analyzed periodically (at least once a week) for size and polydispersity. Particle size and polydispersity index (PdI) were determined by dynamic light scattering (DLS) measurements on a NanoPlus Zeta/nano analyzer (Micrometrics, Georgia, United States). Samples (20 μL) were diluted in 1 mL of purified water. Results are presented as the mean and standard deviation of three independent measurements (n = 3).

### Preparation of the dry powder inhalable microparticles (MPs)

In order to get dry powder MPs by spray-drying method, micronized lactose monohydrate (Lactohale^®^ LH206, DFE Pharma, Germany) was used as a thermic protector for PCL-NP and AZM-PCL-NP dispersions. During NPs preparation (*vide* 2.1.), lactose was solubilized in dilution phase at 0.25, 0.5, 0.75 or 1 wt%. Immediately before evaporation step, the PCL-NP and AZM-PCL-NP dispersions were pumped into a spray-dryer (mini B190, Büchi, Cleveland, United States) filled with a stainless steel standard fluid nozzle tip of 0.5 mm internal diameter. Conditions such as inlet temperature, outlet temperature, atomizing air flow, aspirator air flow and feed rate were investigated (Table 1) to generate the PCL microparticulate powder (PCL-MP) and the AZM loaded powder (AZM-PCL-MP). After spray drying, powder formulations were kept in a desiccator at room temperature. Process yield (%) was calculated with Equation 1, which refers to the weight percentage of the powder collected in relation to the initial weight of the solids dissolved in the feed solution.

$$Process\;yield\;\left(\%\right)=\frac{Amount\;of\;total\;solids\;after\;spray-drying}{Amount\;of\;total\;solids\;before\;spray-drying}\times 100$$ (1)

**TABLE 1:** AZM-PCL-MP formulation yield (%) during the spray-drying process.

Run	Inlet temperature (°C)	Outlet temperature (°C)	Atomizing air flow (LN/h)	Aspirator air flow (%)	Feed rate (mL/min)	Process yield (%)
1	80	57	600	80	5.81	41*
2	80	55	600	70	5.95	38*
3	100	64	600	80	4.72	51
4	100	64	600	70	4.55	63
5	110	54	800	80	7.35	57
6	110	49	800	70	7.27	55
7	120	61	800	80	4.63	58
8	120	59	800	70	4.60	58

*The obtained powder melted inside the spray-drying chamber.

### Physicochemical characterization of the dry powder inhalable microparticles

#### Field emission electron microscopy SEM-FEG

A High-resolution Field Emission Gun Scanning Electron Microscope (FEG-SEM, 3D FEG, Quanta, United States) at 20 kV was used to investigate the microparticle’s morphology. Powder samples (PCL-MPs and AZM-PCL-MPs) were loaded by light dusting onto a metal stub with a double-sided adhesive carbon tape. The samples were coated with palladium (10 nm) with a sputter coater (TN-1100X-SPC-16M, Canada), using an electric potential of 2.0 kV for 2 min. Microscopic images were randomly captured at various magnifications from 500 to 25,000x.

#### Laser light diffraction

The AZM-PCL-MPs and PCL-MPs formulations were resuspended (ca. 0.4 wt%) in distilled water as a dispersing medium, with a refractive index of 1.33. The analyses were carried out in Mastersizer MicroPlus (Malvern, United Kingdom) using a Hydro 2000 G (A) dispersion cell.

#### Differential scanning calorimetry (DSC)

DSC analyzes were carried out using a DSC822 (Mettler-Toledo, Switzerland). Approximately 3–5 mg of the powdered sample (AZM-PCL-MP) and pure excipients were sealed into standard aluminum pans using a pin holed standard aluminum lid. All samples were scanned in the range of 25°C–500°C at a scanning speed of 10°C/min under 50 mL/min nitrogen gas purging using an empty aluminum pan as reference. Thermograms data were collected using the STARe SW thermal analysis software (Mettler Toledo, Switzerland).

#### Thermogravimetric analysis (TGA)

TGA were conducted using a TGA/DSC 1 STARe System (Mettler-Toledo, Switzerland). Samples of approximately 10 mg of AZM-PCL-MPs were loaded into the platinum pan and analyzed in ramp heating mode at 10°C/min from 25°C to 500°C under nitrogen gas purging of 50 mL/min. The percentage of weight variation upon heating was estimated using the Universal Analysis 2000 software (Mettler Toledo, Switzerland).

#### X-ray powder diffraction (XRPD)

The crystallinity of the microparticles was analyzed using an X-ray diffractometer (Rigaku, United States) equipped with a Cu-Kα radiation source and a fixed monochromator. The powder samples of AZM-PCL-MPs were placed in glass holders and X-ray diffraction was measured in the range of 5°–40° 2θ at a scan speed of 5°/min and a step of 0.02°. The voltage and current used were 40 kV and 30 mA, respectively.

#### Attenuated total reflectance and Fourier transform infrared spectroscopy (ATR-FTIR)

ATR-FTIR spectra were acquired using a Nicolet Is10 Spectrometer (ThermoFisher, United States) in the % transmittance mode. The samples (AZM-PCL-MPs and pure excipients) were placed on the ATR diamond crystal with the pressure arm positioned over the sample and spectra were collected at a resolution of 4 cm^−1^ with 64 scans per spectrum over the range 4,000–700 cm^−1^. An unsampled background spectrum was collected before each test. All spectra data were collected using ResolutionPro (5.2.0, Agilent Technologies software).

### Determination of azithromycin loading and encapsulation efficiency

**FIGURE 1:**
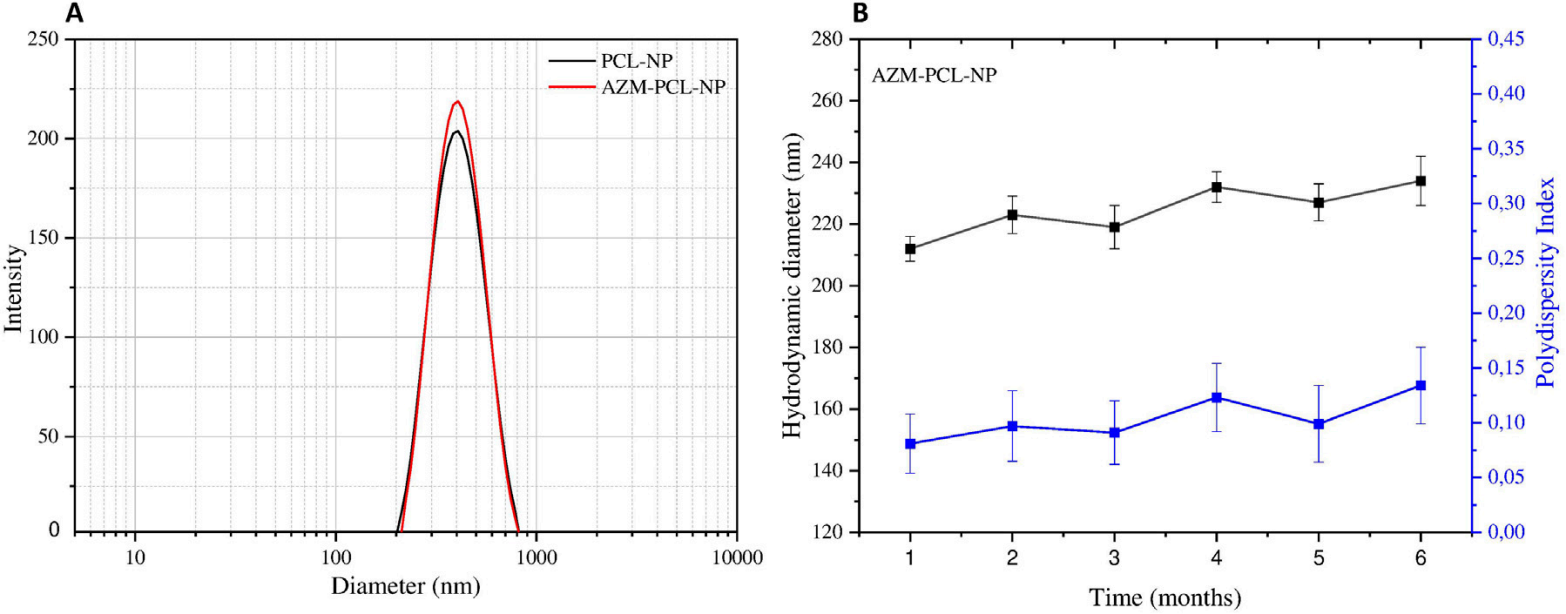
**(A)** Spectrophotometric quantification of azithromycin in buffer solution with pH 7.2 (inset). **(B)** Analytical standard curve of azithromycin quantification in AZM-PCL-MP formulation.

Drug loading and encapsulation efficiency were determined by a modified spectrophotometric method [16, 17]. Briefly, AZM-PCL-MP were dissolved (ca. 0.99 wt%) in 1 mL dimethylsulfoxide (DMSO) and then mixed with 9 mL methanol. After centrifugation at 24,104 × g for 15 min, the supernatant was collected and diluted in PBS (pH 7.2, 50 mM). Then, 5 mL of diluted solution was added to 5 mL of H_2_SO_4_ (13.5 M) and allowed to react for 30 min to produce a yellow color. The solution was analyzed at 482 nm in a spectrophotometer (UV/Vis, M-51 BEL^®^ Engineering Photonics) to determine the AZM concentration based on a standard curve (Figure 1). The encapsulation efficiency (EE%) was calculated with the help of Equation 2 [5].

$$EE\left(\%\right)=\frac{L_{a}}{L_{t}}\times 100$$ (2)

where L_a_ is the total mass of AZM encapsulated and L_t_ is the initial AZM mass added to the system.

### *In vitro* aerosolization performance by next-generation impactor (NGI^®^)

The *in vitro* aerosolization profile of the microparticles was evaluated using a Next-Generation Impactor (NGI^®^, Nottingham, United Kingdom). Size 4 hard gelatin capsules (InfinityPharma, Brazil) were filled with 25 mg of the mixture of AZM-PCL-MPs with micronized lactose LH206 in a 50:50 ratio. Next, the powder capsules were incorporated into the Aerolizer^®^ dry powder inhalation (DPI) device and punctured once. A flow rate of 100L/min was used, which was supplied by two pumps in series (HCP5, UK) and measured using a flow meter (Copley, Nottingham, DFM2000, Copley, United Kingdom). The flow rate generated a pressure drop of 4 KPa across the inhaler device. The execution time for each experiment was set at 2.4 s to simulate *in vivo* inspiration volume (i.e., 4L). The powder collected inside each one of the plates of NGI was dissolved in DMSO and AZM was quantified by UV-Vis. For the NGI flow rate of Q = 100 L/min, the effective aerodynamic cutoff diameter (D_a50_) for each impactor stage was calibrated by the manufacturer and stated as: stage 1, 6.12 µm; stage 2, 3.42 µm; stage 3, 2.18 µm; stage 4, 1.31 µm; stage 5, 0.72 µm; stage 6, 0.40 µm; and stage 7, 0.24 µm. The emitted dose (ED) was determined as the difference between the initial mass of powder loaded in the capsules and the remaining mass of powder in the capsules following aerosolization. The ED (%) (Equation 3) was used to express the percentage of ED based on the total dose (TD) used [18].

$$Emitted\;Dose\;\left(ED\%\right)=\frac{ED}{TD}\times 100\%$$ (3)

The fine particle dose (FPD) was defined as the dose deposited on Stages 2–7 and the fine particle fraction (FPF%) (Equation 4) was expressed as the percentage of FPD to ED.

$$Fine\;Particle\;Fraction\;\left(FPF\%\right)=\frac{FPD}{ED}\times 100\%$$ (4)

The respirable fraction (RF%) Equation 5 was used as the percentage of FPD to total deposited dose (DD) on all impactor stages.

$$Respirable\;Fraction\;\left(RF\%\right)=\frac{FPD}{DD}\times 100\%$$ (5)

Furthermore, the mass median aerodynamic diameter (MMAD) of aerosol particles was determined based on the cumulative particle size distribution functions obtained from the NGI data. The experiment was carried out, filling 5 capsules one by one, manually and containing 25 mg of powder formulation in each, to guarantee an adequate amount for quantification by UV-Vis.

### Cytotoxicity assay

Human bronchial epithelial cell line (BEAS-2B, BCRJ code 0395, Brazil), human fibroblast cell line (HFF-1, ATCC SCRC-1041, United States) and human endothelial cell line (HUVEC, ATCC CRL-1730, United States) were used to investigate the cytotoxicity of AZM, AZM-PCL-NPs and PCL-NPs formulations. The cells were grown in DMEM supplemented with 10% FBS, in an incubator at 37°C, 95% humidity and 5% CO_2_ atmosphere. The cell lines were plated at a concentration of 1.5 × 10^4^ cells/well in a 96-well plate. After incubation for 24 h, the cells were exposed to the AZM or AZM-PCL-NP at different concentrations ranging from 25 μg/mL to 1,600 μg/mL to of AZM for EC50 determination. Cell viability was evaluated based on the MTT (3-(4,5-dimethylthiazol-2-yl)-2,5-diphenyltetrazolium bromide) method based on Mosmann [19]. Briefly, 5 mg/mL MTT solution (Invitrogen, United States) and culture medium at 1:10 ratio were added. The cells were then incubated at 37°C for 3 h. After this time, the medium containing the MTT was removed and 200 µL DMSO was added to solubilize the formazan crystals. Cell viability was determined by absorbance spectroscopy at 595 nm. This assay was performed as 3 independent experiments, each in triplicate. Data analyses were carried out using the Prism 5.0 program and EC50 values were calculated for each cell line studied.

### Antimicrobial activity

The antibacterial efficacy of AZM, AZM-PCL-NP and PCL-NP formulations was evaluated *in vitro* against *Streptococcus pneumoniae* (ATCC 6303) and *Staphylococcus aureus* (ATCC 6538). All microbiological tests were performed in triplicate and results are presented as mean and standard deviation. *S. pneumoniae* strain was maintained in trypticase soy broth (TSB) or trypticase soy agar (TSA) [20] and the *S. aureus* strain in nutrient broth or nutrient agar [21] prepared according to the manufacturer’s instructions. *S. pneumoniae* was kept in microaerophilic environment generation system anaeropack in all tests.

#### Antimicrobial activity by disk diffusion assay

Petri dishes containing nutrient agar and potato-sucrose-agar were seeded with 10^6^ cells of *Staphylococcus aureus* and *Streptococcus pneumoniae*, respectively. In sequence, 6 mm discs impregnated with 20 µL of the respective serial dilutions from 25 to 1,600 μg/mL of AZM, AZM-PCL-NP, PCL-NP were placed in Petri dishes and kept at 37°C for 18 h to analyze bacterial growth and halo formation. Microbiological tests were performed in triplicate [22].

#### Resazurin bacterial susceptibility test

Samples of 10^6^ bacterial cells were plated in 96-well plates and submitted to AZM, AZM-PCL-NP, PCL-NP. The drug concentration ranged from 25 to 1,600 μg/mL and the strains were kept in an incubator at 37°C for 24 h. Two hundred microliters of sterile water was added to all perimeter wells to avoid evaporation during the incubation. After 24 h, 10 µL of resazurin (1 wt%) was added to each well and after 1 h cell viability was observed through color change. A change in color from blue (oxidized state non-viable cell) to pink (i.e., reduced viable cell) indicated bacterial growth [23, 24].

#### Determination of the minimum inhibitory concentration (MIC)

To determine the minimum inhibitory concentration (MIC) of AZM and AZM-PCL-NP formulation, microdilution was performed in 96-well plates containing 10^6^ cells/well of *S. aureus* [20, 25] and *S. pneumoniae*. The AZM concentration ranged from 25 to 1,600 μg/mL. After microdilution in broth, each well with the respective treatment was seeded in Petri dishes with TSA and nutrient agar, for *S. pneumoniae* and *S. aureus*, respectively. After incubation for 24 h at 37°C, the colony forming unit (CFU) count was performed and the minimum concentration required to inhibit bacterial (i.e., CIM) in both strains was calculated.

## Results and discussion

### Production and characterization of AZM-PCL-NPs

#### Dynamic light scattering

**FIGURE 2:**
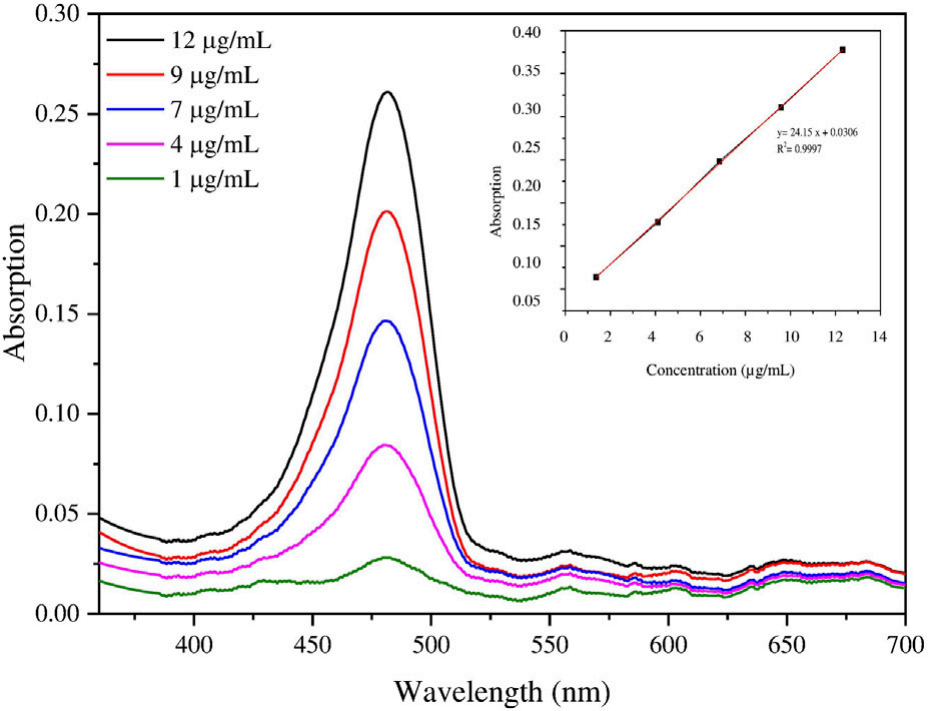
**(A)** Particle size distribution and **(B)** polydispersity index (PdI) and hydrodynamic diameter of AZM-PCL-NPs during 6 months of storage at 4°C based on dynamic light scattering measurements. Results are presented as the mean of three independent measurements (n = 3) plus standard deviation.

The scattering profiles of PCL-NP containing or not AZM are presented in Figure 2A. As can be seen, the placebo PCL-NPs presented mean hydrodynamic diameter of 228 ± 8 nm, while the AZM-loaded formulations (AZM-PCL-NPs) presented mean hydrodynamic diameter around 195 ± 12 nm. The corresponding polydispersity index (PdI) were 0.099 ± 0.047 and 0.137 ± 0.032, respectively, indicating homogenous monodisperse systems. These values are similar to previous ones reported in the literature for PCL nanoparticles prepared by the emulsion/solvent diffusion-evaporation method, corresponding to structures of 180–250 nm [26]. The small size and narrow size distribution can be related to the NP preparation method, such as emulsification-diffusion-evaporation. The ratio between components of the organic phase, high shear speed (i.e., 14,000 rpm) promotes the breakage of droplets into smaller ones, resulting in a narrow size distribution [27]. The use of surfactant copolymer (i.e., Pluronic F-127) stabilizes the interface between the organic and aqueous phase, preventing droplet coalescence during emulsification process. Maintaining the aqueous phase in excess can favor the efficient diffusion of the solvent into the aqueous phase, facilitating the formation of homogenous NPs. Finally, conducting the diffusion and evaporation processes at low temperature (i.e., 50°C) allows the formation of more uniform NPs [28].

This approach also allowed us to achieve a high AZM encapsulation efficiency (%EE) of 83%. The adequate molar proportion of AZM, PCL and DPPC (0.06:0.19:0.75) in the AZM-PCL-NP formulation favored the formation of homogeneous and stable droplets, avoiding particle aggregation and promoting better drug retention inside the NPs, resulting in a high EE%. The adequate ratio of AZM:PCL provided the necessary polymeric matrix to encapsulate AZM, while DPPC stabilized the particle structure without compromising encapsulation. Besides, Pluronic F127 used as a polymeric surfactant also stabilized the emulsification and diffusion process, preventing particle coalescence and resulting in homogeneous particles, favoring high %EE [29, 30]. A high drug encapsulation efficiency of 84% for paclitaxel was also achieved using PLGA-PCL microparticles [31].

### Production of PCL-MP and AZM-PCL-MP inhalable powders

Spray-drying is a versatile process to dry polymeric nanostructures and parameters such as inlet temperature, atomizing air flow, aspirator air flow and consequently the feed rate, can be studied [15]. Usually, inlet temperatures from 120°C to 125°C are used [17], nevertheless inlet temperatures from 80–120°C were also previously tested and resulted in compact and homogenous powders [32].

Regarding the use of micronized lactose LH206 as a drying adjuvant, the lactose melted inside the spray-dryer when used at concentrations between 0.25% and 0.75%, probably because the inlet temperatures was too high for this concentration. However, at 1%, lactose contributed to a more stable formulation, producing a homogeneous powder, probably due to the formation of a film around the NPs.

As can be seen in Table 1, run 1 and run 2 resulted in the lowest yields, owing to the higher deposition of the powder on the cyclone walls that was apparent during the production process. The best yield of 63% was obtained for run 4 and corresponded to a higher outlet temperature of 64°C, inlet temperature of 100°C, atomizing air flow in 600 NL/h, aspiration air flow in 70% and feed rate of 4.55 mL/min, which resulted in a homogenous white powder. Inlet temperature at 100°C and the outlet temperature around 64°C, can help prevent AZM degradation while ensuring proper water evaporation. For PCL, these inlet/outlet temperatures allowed it to remain in a solid state, ensuring that the microparticles do not coalesce or lose their structural integrity during drying [33].

### Characterization of the AZM-PCL-MP inhalable powder

#### Thermal analysis (DSC/TGA)

**FIGURE 3:**
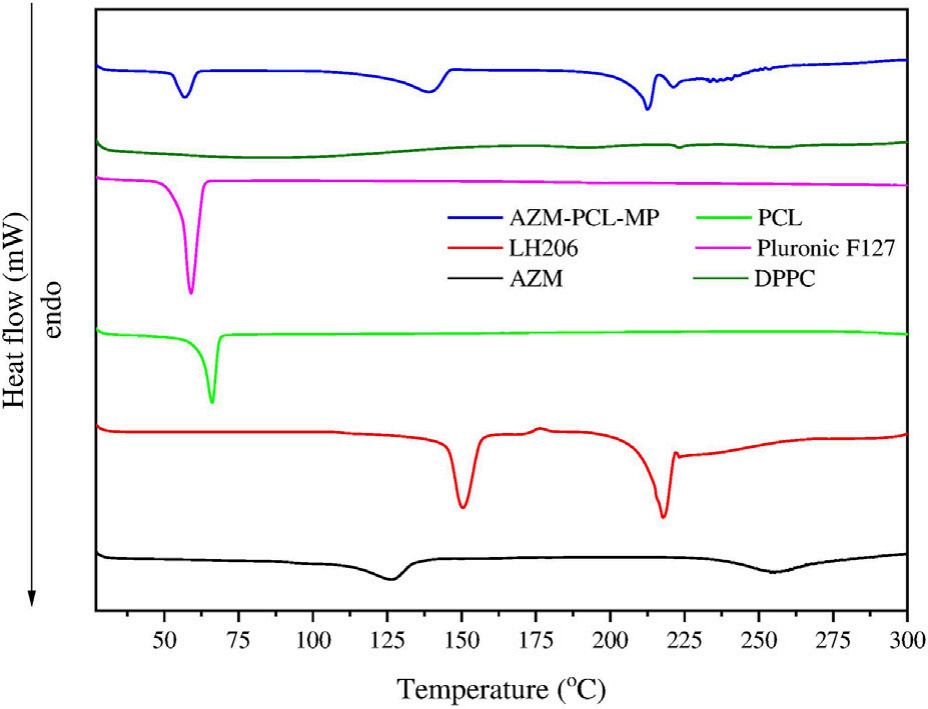
Differential scanning calorimetry (DSC) thermograms of azithromycin (AZM), lactose LH206, polycaprolactone (PCL), PluronicF127, dipalmitoylphosphatidylcholine (DPPC) and microparticles after spray-drying (AZM-PCL-MP).

DSC analysis was performed on the raw materials (AZM and excipients) as well the AZM-PCL-MPs to confirm the successful loading of the drug into the formulation (Figure 3). According to previous reports, the melting temperature (T_m_) of PCL was demonstrated to be 60°C–71°C [34, 35]. In this study, the endothermic peak of PCL was observed at 66°C. The T_m_ value of Pluronic F127 obtained in this work, 57°C, is similar to that reported by Karolewicz et al. [36]. For DPPC, no thermal event was observed in the range of 25°C–300°C, which has already been verified by Eedara et al. [37]. On the other hand, the lactose LH206 presented the T_m_ at 221°C. Listiohadi et al. [38] reported another thermodynamic event of exothermic nature for lactose in the range of 150°C–175°C, which suggests a possible recrystallization of the amorphous phase. We observed this transition around 176°C. AZM presented a T_m_ value of 126.4°C, which is also closer to the value reported by Maswadeh et al. [39] at 125°C. Furthermore, another thermodynamic event can be observed around 260°C, which can be attributed to the AZM degradation temperature (Td).

For the AZM-PCL-MP (Figure 3), the endothermic peak shifted to a lower temperature of 57°C, corresponding to the T_m_ of PCL and Pluronic F127 and indicating the miscibility of both polymers in the formulation. Endothermic events at 132°C and 212°C can be attributed to lactose monohydrate, present in higher proportion in the formulation. The endothermic peak corresponding to the Tm of AZM (126°C) was not observed, indicating that the AZM is dispersed in the polymeric nanoparticle matrix [33]. Contributions from the NP formation process may result in the absence of the AZM melting peak, such as the interactions between AZM and the PCL matrix, which stabilize the drug in the amorphous form [40]. The spray-drying process also promotes rapid drying of NPs, preventing recrystallization of AZM. Lactose, which acts as a drying aid and can improve the distribution of AZM in the matrix [41]. Therefore, the resulting formulation presents a molecular or solid dispersion of AZM, which explains the absence of the AZM melting peak in the DSC analysis.

**FIGURE 4:**
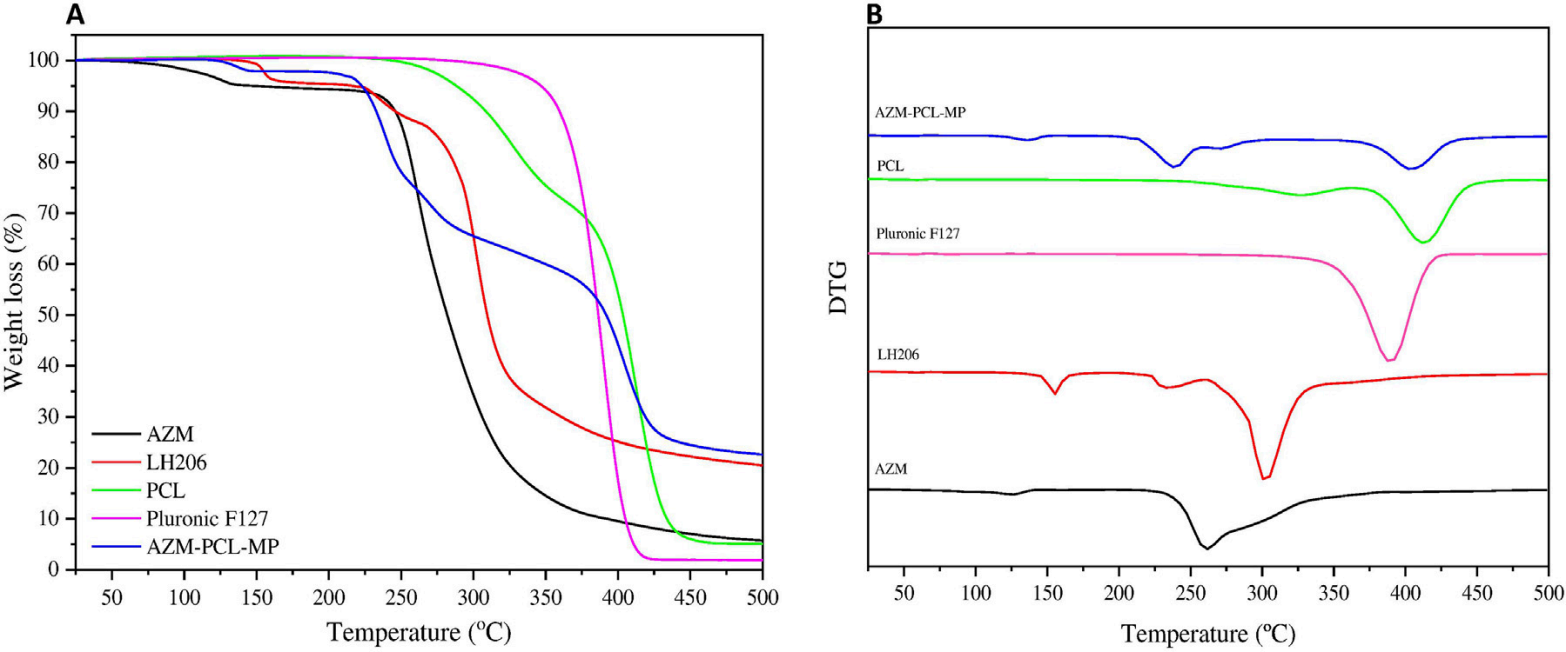
**(A)** Thermogravimetric analysis (TGA) and **(B)** DTG curves of azithromycin (AZM), lactose monohydrate (LH206), polycaprolactone (PCL), pluronicF127 and dried AZM-PCL-MP formulation.

The TGA curves (Figure 4A) represent the thermal degradation of the components (AZM and excipients) and the spray-dried AZM-PCL-MPs. The PCL thermogram showed two events of degradation at 325°C and 410°C. Pluronic F127 thermograms showed one-step process of thermal decomposition at 389°C, corroborating with previous results [33]. AZM presented thermal degradation at 260°C, reaffirming results of DSC analysis [37]. The TGA curve for LH206 showed a minimum loss of mass (ca. 0.4%) between 40°C and 130°C which corresponds to the loss of water at the surface, while a second mass loss (ca. 5.1%) around 153°C, indicating a loss of hydration water [42]. The TGA curve for spray-dried AZM-PCL-MPs remained constant up to 120°C where loss of water was observed. A second event occurs at 212°C, leading to an abrupt drop in the mass (ca. 40%) and suggesting the beginning of the decomposition. Finally, a decomposition of more than 70% is observed at 400°C.

The Derivative Thermogravimetry (DTG) curves of raw materials (AZM and excipients) and spray-dried AZM-PCL-MPs are shown in (Figure 4B). The thermal stability of PCL and Pluronic F127 were observed in the thermal degradation pattern of the spray-dried formulation AZM-PCL-MP indicating that both polymers retained their original thermal behavior in the microparticles. PCL and Pluronic F127 showed a clear degradation peak temperature at 410°C and 388°C, respectively, confirmed by Ramírez-Agudelo et al. [43]. AZM thermogram showed a thermal degradation at 260°C, in agreement with values reported by Maswadeh et al. [37]. According to the DTG results, the thermal degradation of AZM-PCL-MP could be divided into three stages. The first stage is associated to the intermolecular water loss around 136°C. The second stage at 220°C is attributed at the degradation of LH206, which is in higher proportion in the AZM-PCL-MP formulation. The third stage is assigned to the decomposition of the AZM-PCL-MP formulation at 400°C. Data obtained by DSC not only corroborate with the TGA, but also present first order phase transitions (melting temperature), indicating the high degree of purity of the microparticles produced.

#### X-ray powder diffraction (XRPD)

**FIGURE 5:**
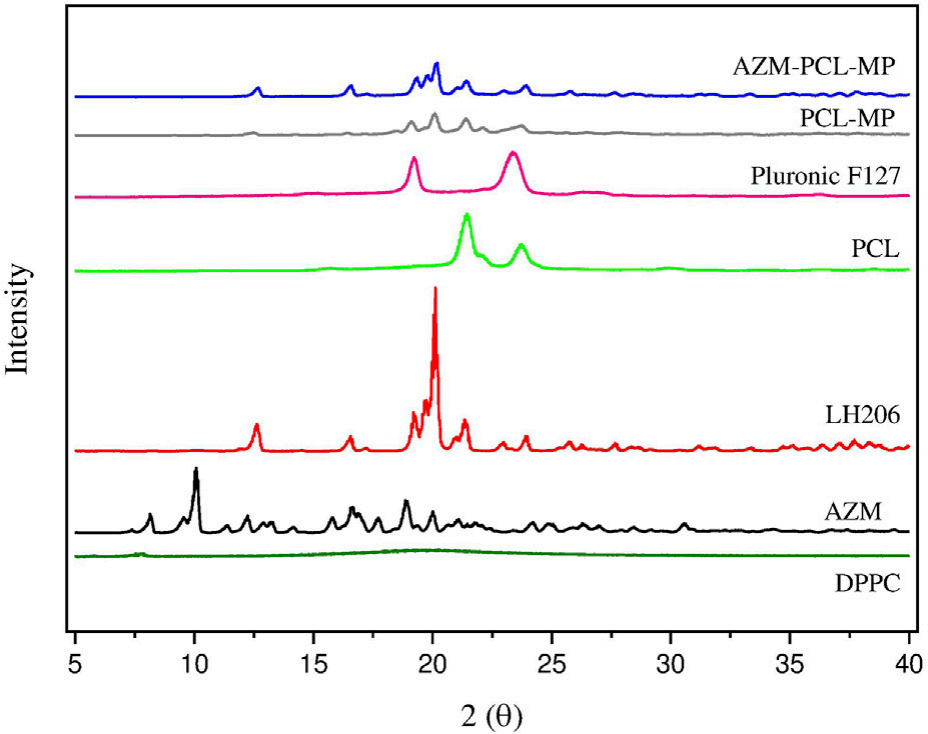
X-ray diffraction of azithromycin (AZM), lactose LH206, polycaprolactone (PCL), PluronicF127, dipalmitoylphosphatidylcholine (DPPC) and spray-dried formulations (PCL-MPs and AZM-PCL-MPs).

X-ray powder diffraction was used to evaluate the solid-state properties of both raw materials (AZM and excipients) and spray-dried AZM-PCL-MPs (Figure 5). Data on the crystal lattice parameters of AZM have already been described in the literature [44]. AZM at room temperature is an orthorhombic structure, and belongs to the space group P21, a = 14.735 Å, b = 16.844 Å, c = 17.810 Å, α = 90°, β = 90°, γ = 90°, describing the crystallinity of AZM.

In this work, the AZM diffractogram showed an intense peak at 10.06° (2θ) and other sharp peaks at 16.62°, 17.80°, 18.88° and 19.96° (2θ), highlighting the crystallinity of the drug as studied by [5], where similar peaks were obtained for AZM encapsulated by poly-lactic acid (PLA). PCL and Pluronic F127 exhibited the characteristics of a semicrystalline polymer and copolymer, respectively, with two broad peaks at 21.36°; 23.78° and 19.28°; 23.46° (2θ), respectively. These peaks were also found for other research using PCL and chitosan nanofibers with encapsulated AZM [40]. Lactose LH206 appeared as a crystalline material, with an intense peak at 20.12° 2θ and the presence of some peaks with lower intensity at 12.68°, 19.28° and 21.38° (2θ). On the other hand, in the DPPC, a diffraction pattern without peaks and in a disorganized form can be observed, demonstrating the amorphous structure of this raw material.

For PCL-MPs and AZM-PCL-MPs, they showed a peak with greater intensity at 20° and 20.1° (2θ), and other peaks with lower intensity were observed at 12.5° and 19.2° (2θ), which are attributed to lactose LH206. Furthermore, both powder formulations (PCL-MPs and AZM-PCL-MPs) exhibited diffraction patterns similar to those obtained for PCL (21.48° 2θ) and Pluronic F127 (23.1° 2θ) raw materials. However, no peaks were observed for AZM (10.06° 2θ), indicating that AZM is preferably dispersed in the polymeric nanoparticle matrix [33].

#### ATR-FTIR measurements

**FIGURE 6:**
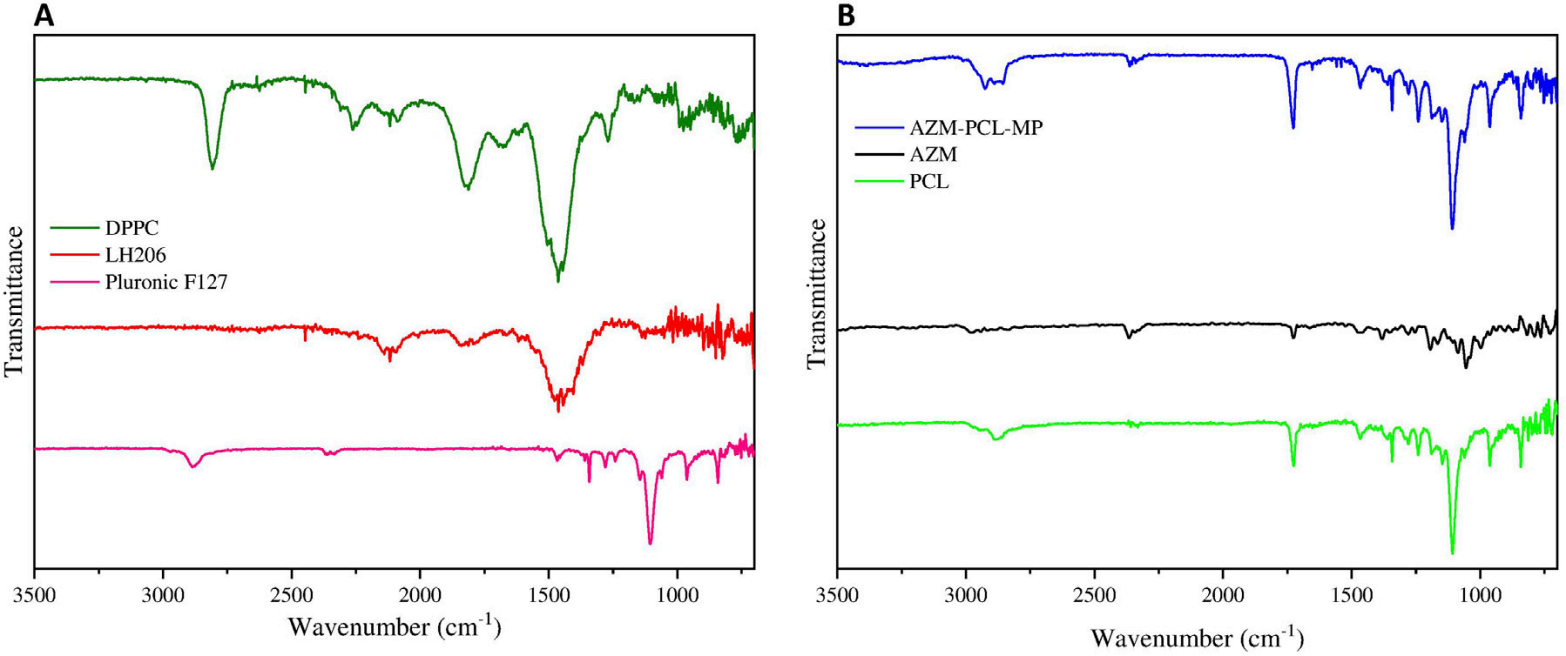
**(A)** FTIR spectrum of Pluronic F127, lactose monohydrate (LH206), phospholipid (DPPC), **(B)** FTIR spectrum of PCL, AZM and spray-dried AZM-PCL-MP formulation.

According to (Figure 6A, the spectrum of Pluronic F127, showed the main stretching of aliphatic bands (C-H) around 2,882 cm^−1^ and symmetric C-O-C stretching at 1,104 cm^−1^, already reported in literature [45]. Lactose LH206 showed the stretching vibration of the hydroxyl group (OH) at 3,667 cm^−1^ and the characteristic bands at 2,989 cm^−1^ and 2,898 cm^−1^ attributed to the asymmetric and symmetric stretching vibrations of the CH_2_, respectively [46]. The FTIR spectrum of DPPC presented the principal band between 3,600 cm^−1^ and 3,700 cm^−1^, which indicates the vibration of the OH group. Asymmetric and symmetric vibration at 2,931 cm^−1^ and 2,852 cm^−1^ were observed and attributed to the CH_2_ group. The band at 1736 cm^−1^ clearly represents the carbonyl stretching. The stretching vibrations of the phosphate group were observed in the range of 1,230 cm^−1^ and 1,190 cm^−1^. In addition, the band at 1,050 cm^−1^ refers to the vibrations of the CO-PO_2_ group vibrations [47].

In Figure 6B, the spectrum of AZM showed a band in the range of 3,559 cm^−1^ and 3,488 cm^−1^, attributed to the vibration of N-H group [37]. Furthermore, The CH_2_ groups were also confirmed by the asymmetric and symmetric vibrations bands at 2,974 cm^−1^ and 2,898 cm^−1^, respectively [48]. The band at 1,721 cm^−1^ confirmed the vibration of the carbonyl group. Other bands between 1,123 cm^−1^ and 1,250 cm^−1^ suggested the absorption associated with axial stretching of the C-O functional group [49].

The FTIR spectrum of PCL (Figure 6B) has a characteristic band at 2,950 cm^−1^ indicating the asymmetric CH_2_ stretching and symmetric CH_2_ stretching at 2,880 cm^−1^. The band at 1,723 cm^−1^ represents the carbonyl stretching and the 1,108 cm^−1^ was attributed to the symmetric C-O-C stretching [50].

Comparing the FTIR spectra of the raw materials and the AZM-PCL-MP formulation (Figures 6A, B), we can see that the characteristic band of AZM was present in the formulation spectra. For AZM-PCL-MPs (Figure 6B) spectra, not only the carbonyl group at 1,725 cm^−1^ but also the asymmetric and symmetric stretching vibrations of CH_2_ at 2,931 cm^−1^ and 2,852 cm^−1^ were observed. The band at 1,100 cm^−1^corresponds to vibrations of C-O-C and were expected according to the raw materials. In addition, the displacement of the bands corresponding to the AZM for AZM-PCL-MPs indicated the formation of microparticles in which the drug was molecularly dispersed [5].

#### Particle size distribution

**FIGURE 7:**
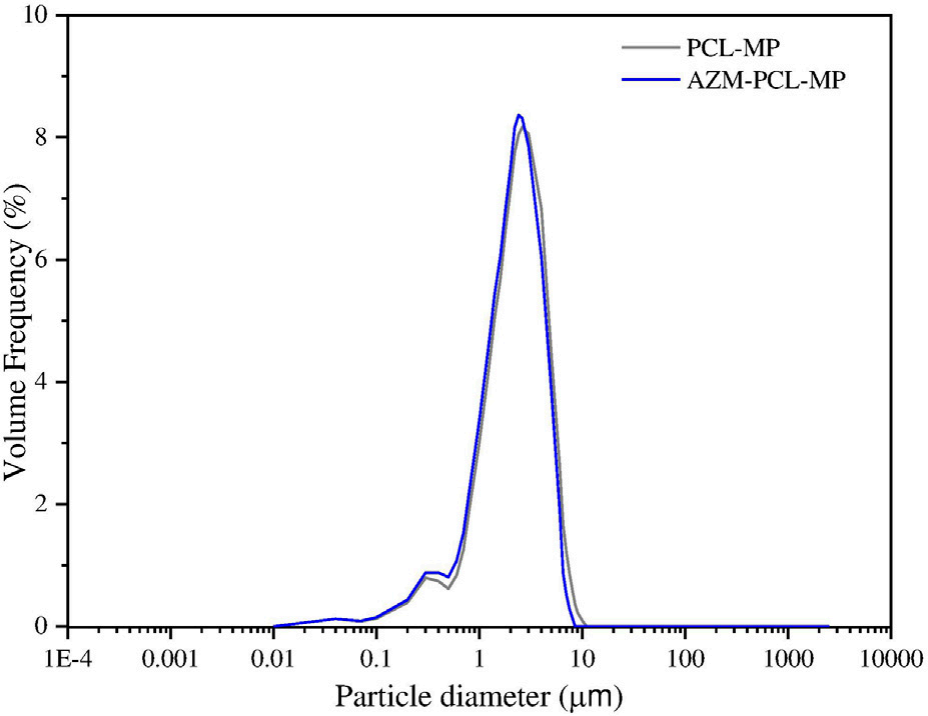
Particle size distribution by laser diffraction of spray-dried; PCL-MP and AZM-PCL-MP formulations.

The particle size distribution of PCL-MP and AZM-PCL-MP formulations are showed in Figure 7. For the deep lung deposition of inhalable dry powders, diameters in the range of 1–5 µm are required [51]. In this study, 90% (d_90_) of the AZM-PCL-MPs presented a size of 4.06 µm, or smaller which may result in successful deposition in the deeper airways. This particle size distribution can be related to the lower concentration of PCL used during the emulsification process, which consequently results in smaller microparticles when dried by spray-drying process. When the PCL concentration is reduced in the emulsification step, the viscosity of the organic phase decreases, leading to a smaller droplet size due to more efficient dispersion and breakup during emulsification step. This effect is consistent with observations made by Balmayor et al., who reported that lower PCL concentrations lead to a decrease in the size of microparticles formed during the emulsification process. In the spray-drying process, rapid solvent evaporation solidifies the droplets, fixing the microparticle size [52]. Since PCL has a relatively low glass transition temperature and is flexible, it supports the formation of small, spherical particles when processed under optimal conditions [53].

The particle size distribution of powder formulation is summarized in Table 2. As can be seen, volume median diameter ( $d_{0.5}$ ) and volume mean diameter ( $d_{4.3}$ ) varied from 1.95 µm to 1.96 µm and 2.47 µm to 2.83 µm, respectively. Span values of 1.70–1.83 indicate homogenous size distributions, which is important for accurate dosing [54].

**TABLE 2:** Particle size of spray-dried formulations.

	Particle size distribution^a^				
Sample formulation	$d_{0.1}$ (µm)	$d_{0.5}$ (µm)	$d_{0.9}$ (µm)	$d_{4.3}$ (µm)^b^	Span
PCL-MP	0.46 ± 0.03	1.95 ± 0.01	4.03 ± 0.33	2.47	1.83
AZM-PCL-MP	0.72 ± 0.03	1.96 ± 0.01	4.06 ± 0.49	2.86	1.70

Data are means ± SD (n = 3 independent measurements).

Mean ± standard deviation (SD).

^a^ Equivalent volume diameters at 10% ( $d_{0.1}$ ), 50% ( $d_{0.5}$ ), and 90% ( $d_{0.9}$ ) cumulative volume.

^b^ Volume mean diameter.

#### Particle morphology

**FIGURE 8:**
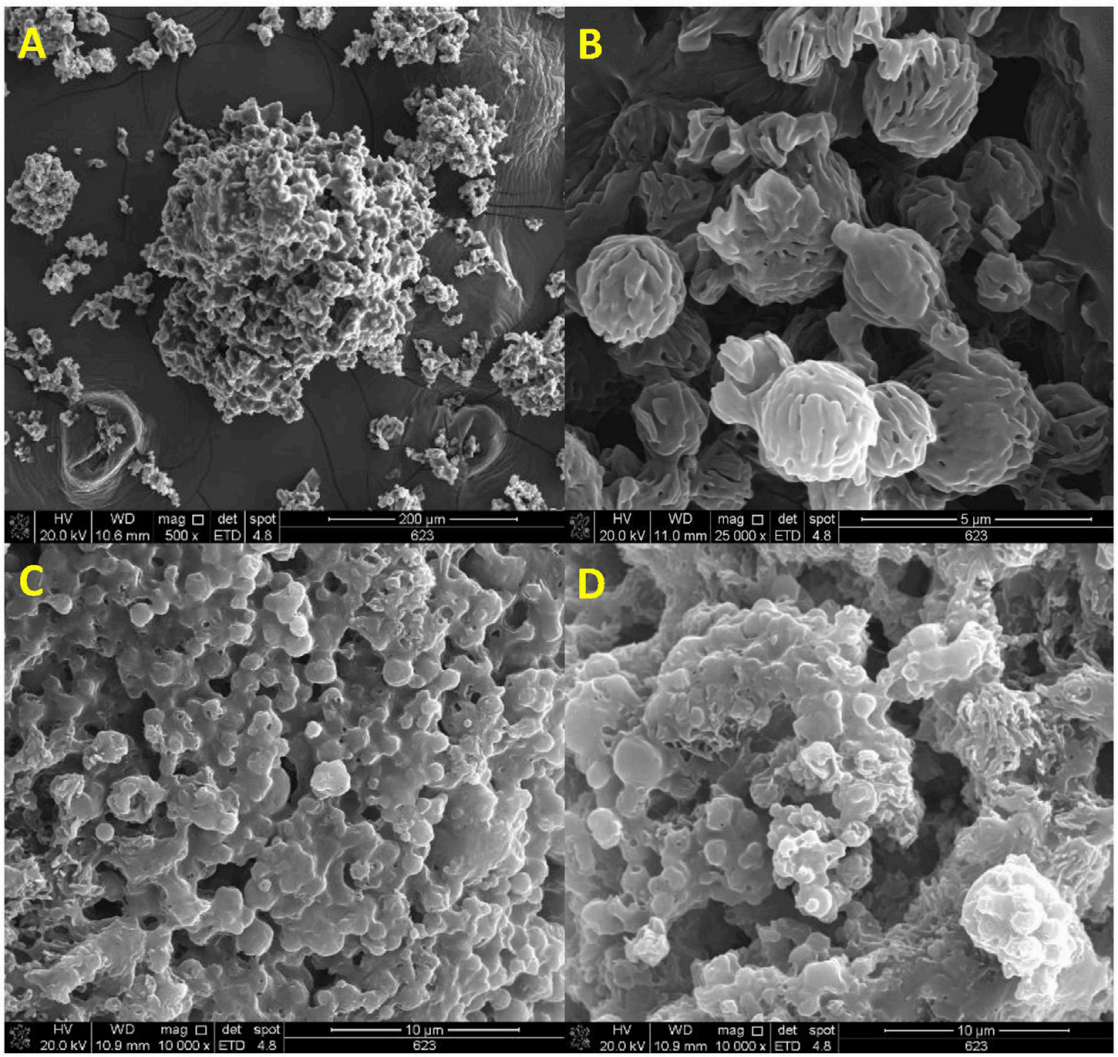
SEM micrographs of **(A)** azithromycin (AZM), **(B)** spray-dried lactose LH206, **(C)** spray-dried AZM-PCL-MP formulation, **(D)** spray-dried PCL-MP formulation.

The morphology of AZM, lactose LH206 and microparticles was investigated by SEM (Figure 8A) exhibited a slightly rough surface, in an aggregated disposition, with D > 10 µm. Spherical microparticles smaller than 5 µm and presenting a rough and highly porous surface were observed in lactose after spray-drying (Figure 8B).

Notably, the obtained AZM-PCL-MPs and PCL-MPs present a nearly spherical shape < 5 µm (Figures 8C, D). The microparticles presented shapeless surface, as expected for a complex mixture including DPPC phospholipids, polymers (PCL and Pluronic F127), AZM and lactose LH206. Other authors showed uneven particles and aggregation when adding different types of drying aids such as mannitol or leucine into polymeric formulations [32]. Rough surfaces were predominantly observed in our spray-dried formulations, which is preferred for pulmonary delivery since it tends to increase the aerosolization efficiency [49].

Overall, the particles obtained in this work showed a proper powder dispersion, which can result in an optimal inhalation profile and, therefore, higher drug delivery into the deeper airways [55].

### Aerodynamic performance by NGI

Microparticles for oral inhalation must have adequate aerodynamic behavior. Therefore, the aerodynamic properties of AZM and AZM-PCL-MPs were characterized using an NGI. AZM-PCL-MPs was deposited successfully in all stages (1–7), while AZM was deposited just at the first stages (1–3). As seen in Table 3, mass median aerodynamic diameter (MMAD) values are remarkably different for AZM and AZM-PCL-MP, 12.34 µm and 5.13 µm, respectively, and MMAD values between 1 and 5 µm are preferable for reaching depth in the lungs. These results are similar to other inhalable dry powders prepared by conventional spray-drying technique [6]. According to the aerosol dispersion performance parameters, AZM-PCL-MP, displayed a fine particle fraction (FPF) around 55% indicating good aerosolization characteristics and suitability for oral inhalation and lung delivery. In contrast, the AZM presented lower values of ED, FPF and RF when compared with AZM-PCL-MP, which we attributed to the aggregates and higher aerodynamic diameter of AZM (ca. 12 μm), leading to reduced FPF and RF values.

**TABLE 3:** Aerodynamic properties of AZM and AZM-PCL-MPs determined by next-generation impactor (NGI) including emitted dose (ED, %), fine particle fraction (FPF, %), respirable fraction (RF, %) and Mass Median Aerodynamic Diameter (MMAD, µm).

Powder	Aerosol dispersion performance parameters			
	ED (%)	FPF (%)	RF (%)	MMAD (µm)
AZM	44	15	8	12.32
AZM-PCL-MP	87	55	42	5.13

### Cytotoxicity assay

**FIGURE 9:**
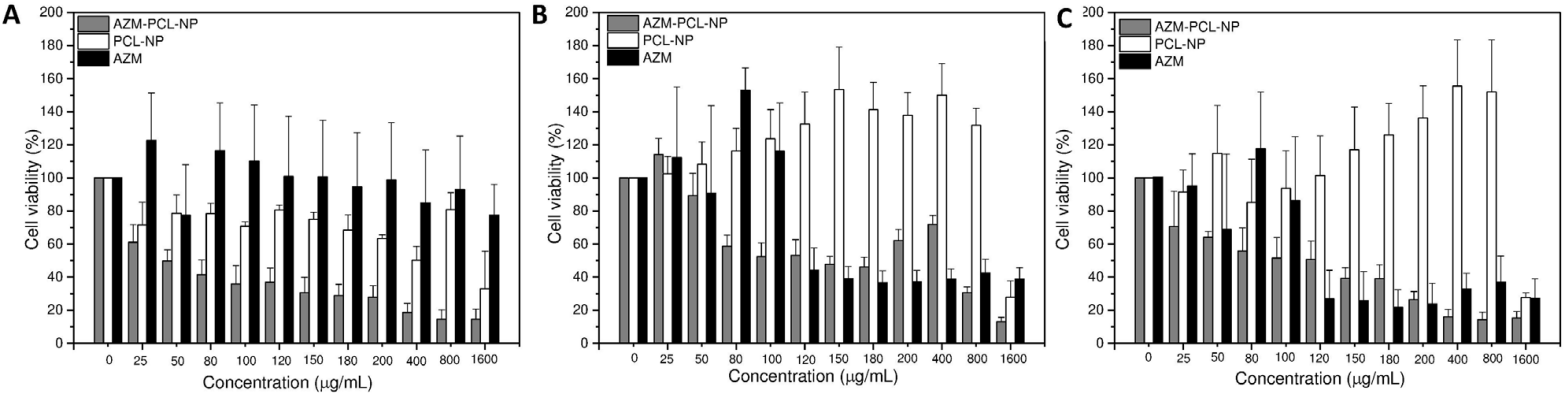
Cell viability assay of AZM, AZM-PCL-NP and PCL-NP formulations in **(A)** BEAS-2B cells, **(B)** HUVEC cells and **(C)** HFF-1 cells.

Since the microparticles can be inhaled orally, they may directly interact with lung cells. As primary assessment, we used MTT assay to verify whether our formulations and its components trigger cellular toxicity. Consequently, human cell lines BEAS-2B (lung epithelial), HUVEC (endothelial) and HFF1 (fibroblasts) were exposed to a wide range of AZM concentrations (25–1,600 μg/mL) either free or incorporated into nanoparticles during 24 h. The results of the MTT assay are summarized in (Figure 9). As can be seen, HUVEC (Figure 9B) and HFF-1 (Figure 9C) were more susceptible to the free AZM with 30%–40% of cell viability compared to BEAS-2B cells (Figure 9A) with 80–100% of cell viability at the highest concentrations (120–1,600 μg/mL). Rampacci et al. [56] also demonstrated a dose-dependent viability of BEAS-2B cells to AZM.

The placebo PCL-NPs showed very compatible with HUVEC (Figure 9B) and HFF-1 (Figure 9C) cells, with viability around 100% at concentrations up to 800 μg/mL. BEAS-2B cells (Figure 9A), on the other hand, presented some sensitivity to the PCL-NPs, nonetheless up to 70% of cell viability was preserved at concentrations up to 150 μg/mL. Thus, the good biocompatibility of PCL makes this biopolymer an appropriate carrier for drug delivery [47].

For AZM-PCL-NPs toxicity higher than 40% was already observed for 25 μg/mL were BEAS-2B cells (Figure 9A), indicating the nanostructures might have promoted the intracellular drug delivery. The same effect was observed in HUVEC (Figure 9B) and HFF-1 (Figure 9C) for concentrations up to 100 μg/mL of AZM loaded in the PCL-NPs. Above this concentration, the AZM was found to be in general more toxic than the AZM-PCL-NPs. Therefore, there should be a balance between the drug and the nanostructures toxicity.

### Antibacterial activity

Halos formation by *S. aureus* and *S. Pneumoniae* were observed at all concentrations tested for AZM-PCL-NP and AZM (Table 4). Halos below 13 mm indicate resistance and halos ≥13 mm indicate sensibility to AZM. As can be seen in (Table 4), halos ≥18 mm were formed in *S. aureus* and *S. Pneumoniae* in the presence of AZM-PCL-NP. No significant differences were observed between the halos formed with AZM and AZM-PCL-NP, therefore both strains could in principle be adequately treated with AZM-PCL-NP.

**TABLE 4:** Antimicrobial activity for AZM and AZM-PCL-NPs disk diffusion test against *S. aureus* and *S. pneumoniae*.

AZM concentration (µg/mL)	*S. aureus*		S. pneumoniae	
	AZM-PCL-NP D_Halo_ (mm)	AZM D_Halo_ (mm)	AZM-PCL-NP D_Halo_ (mm)	AZM DHalo (mm)
1,600	26.0 ± 1.0	25.0 ± 1.0	38.0 ± 2.0	36.0 ± 0.0
800	24.7 ± 0.6	24.0 ± 1.0	35.0 ± 1.0	34.7 ± 0.6
400	23.0 ± 1.0	21.0 ± 0.0	31.7 ± 0.6	31.0 ± 2.0
200	22.0 ± 1.0	20.3 ± 0.6	28.3 ± 0.6	29.3 ± 0.6
100	19.0 ± 1.0	19.3 ± 0.6	25.3 ± 0.6	25.0 ± 1.0
50	18.0 ± 1.0	16.7 ± 0.6	24.0 ± 2.0	22.0 ± 2.0

D_halo_ = diameter of inhibition halo. Mean ± standard deviation (SD).

Nanoparticles can improve drug permeability across the cell membrane enhancing intracellular accumulation, antibacterial activity against the resistant strains, offering multiple bactericidal mechanisms, and inhibiting the biofilm formation by *S. aureus* [57]. Thus, the AZM-PCL-NP delivery system we developed presents strong potential to overcome the challenges associated to the treatment of *S. aureus* and *S. pneumoniae* infections.

### Bacterial viability

After demonstrating growth inhibition of *S. aureus* and *S. pneumoniae*, the antibacterial effect of AZM, either free or incorporated into nanoparticles formulations was evaluated by resazurin bacterial susceptibility test. The resazurin reduction assay depends on the ability of metabolically active cells to reduce the resazurin redox dye to resorufin and is used to determine viability in bacterial cells [58]. After 24 h of each treatment and 1 h of incubation with resazurin, non-viable cells were observed, with an oxidized state through the detection of the blue color reaching the same results both in *S. aureus* and in *S. pneumoniae* bacteria (Table 5).

**TABLE 5:** Bacterial viability test of AZM, PLC-NP and AZM-PCL-NP formulations in *S. aureus* and *S. pneumoniae*.

AZM (µg/mL)	*S. aureus*			*S. pneumoniae*			
	AZM-PCL-NP	AZM	PCL-NP	AZM-PCL-NP	AZM	PCL-NP	
0	V	V	V	V	V	V	
50	NV	NV	NV	NV	NV	NV	
100	NV	NV	V	NV	NV	V	
200	NV	NV	V	NV	NV	V	
400	NV	NV	V	NV	NV	V	
800	NV	NV	V	NV	NV	V	
1,600	NV	NV	V	NV	NV	V	

V: Viable cell (reduced)/NV: Non-viable cell (oxidized state).

Finally, the minimum concentration required to inhibit growth (MIC) of both strains *S. aureus* and *S. pneumoniae* was found to be 3 μg/mL of AZM-PCL-NPs. No cell growth was observed after treatment with free AZM. On the other hand, bacteria with placebo (PCL-NPs) resulted in colony formation.

## Conclusion

In this work, spherical and homogenous AZM-loaded PCL nanoparticles functionalized with DPPC were produced by the emulsion/solvent diffusion-evaporation method. Our formulation preserves the antimicrobial effect of AZM against *S. aureus* and *S. pneumoniae*, two important pathogens of the respiratory tract. Besides, the formulation did not trigger acute cytotoxicity to human endothelial, pulmonary, and fibroblast cell lines. The colloidal dispersion (AZM-PCL-NP) was stable at 4°C up to 6 months in terms of small hydrodynamic diameter and narrow monomodal size distribution and was further used to prepare a dry powder of adequate size for pulmonary delivery through spray-drying. The aerosolizable microparticles formulation (AZM-PCL-MP) was obtained as a white homogenous powder of adequate size for pulmonary delivery with optimal aerosolization properties. Also, the dry powder formulation was characterized by thermal analysis and no degradation or reaction of the components was observed at room temperature. Thus, the AZM-PCL-MPs developed present optimized physicochemical properties that can ensure the successful aerosolization and deposition of dry powder in the lungs and, therefore, can be tested as a novel platform to deliver antibiotics for lung infections. In summary, we produced a potential alternative to treat respiratory tract infections by inhalation therapy.

## Data Availability

The original contributions presented in the study are included in the article/supplementary material, further inquiries can be directed to the corresponding authors.
